# 2,3-Dihydro-1*H*-pyrrolizin-1-one

**DOI:** 10.1107/S1600536810034902

**Published:** 2010-09-25

**Authors:** Yousaf Ali, Yu Peng, Erbing Hua, Mohammad Aijaz Anwar, Mehboob Ali Kalhoro

**Affiliations:** a Pharmaceutical Research Center, PCSIR Laboratories Complex, Karachi 75280, Pakistan; b Department of Pharmaceutical Engineering, Biotechnology College, Tianjin University of Science and Technology, Tianjin 300457, People’s Republic of China

## Abstract

There are two nearly identical mol­ecules in the asymmetric unit of the title compound, C_7_H_7_NO. The mol­ecules are nearly planar (r.m.s. deviations of 0.025 and 0.017 Å) and oriented at a dihedral angle of 28.98 (3)°. The two mol­ecules are linked by a C—H⋯O hydrogen bond. In the crystal, weak inter­molecular C—H⋯O hydrogen bonds link the mol­ecules into zigzag chains along the *c* axis.

## Related literature

For general background to 2,3-dihydro­pyrrolizine derivatives and their biological activity, see: Skvortsov & Astakhova (1992 ▶). For the preparation, see: Braunholtz *et al.* (1962 ▶); Clemo & Ramage (1931 ▶). For natural sources, see: Meinwald & Meinwald (1965 ▶).
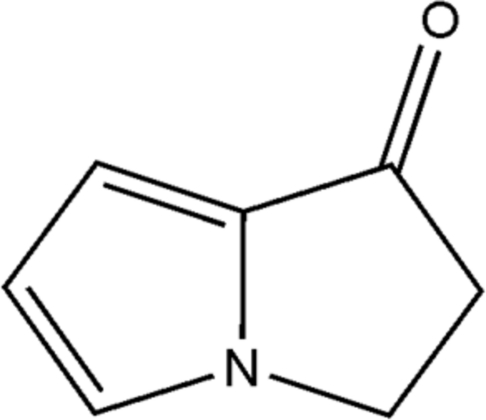


## Experimental

### 

#### Crystal data

C_7_H_7_NO
*M* *_r_* = 121.14
Monoclinic, 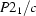

*a* = 11.301 (1) Å
*b* = 7.1730 (7) Å
*c* = 14.3760 (16) Å
β = 90.989 (5)°
*V* = 1165.2 (2) Å^3^
*Z* = 8
Mo *K*α radiation
μ = 0.09 mm^−1^
*T* = 113 K
0.12 × 0.06 × 0.04 mm

#### Data collection

Rigaku Saturn724 CCD camera diffractometer
Absorption correction: multi-scan (*CrystalClear-SM Expert*; Rigaku, 2009 ▶) *T*_min_ = 0.989, *T*_max_ = 0.996
10183 measured reflections
2284 independent reflections
2003 reflections with *I* > 2σ(*I*)
*R*_int_ = 0.051

#### Refinement

*R*[*F* ^2^ > 2σ(*F* ^2^)] = 0.057
*wR*(*F* ^2^) = 0.118
*S* = 1.16
2284 reflections
163 parameters
H-atom parameters constrained
Δρ_max_ = 0.19 e Å^−3^
Δρ_min_ = −0.30 e Å^−3^

### 

Data collection: *CrystalClear-SM Expert* (Rigaku, 2009 ▶); cell refinement: *CrystalClear-SM Expert*; data reduction: *CrystalClear-SM Expert*; program(s) used to solve structure: *SHELXS97* (Sheldrick, 2008 ▶); program(s) used to refine structure: *SHELXL97* (Sheldrick, 2008 ▶); molecular graphics: *ORTEP-3* (Farrugia, 1997 ▶); software used to prepare material for publication: *publCIF* (Westrip, 2010 ▶).

## Supplementary Material

Crystal structure: contains datablocks I, global. DOI: 10.1107/S1600536810034902/bq2209sup1.cif

Structure factors: contains datablocks I. DOI: 10.1107/S1600536810034902/bq2209Isup2.hkl

Additional supplementary materials:  crystallographic information; 3D view; checkCIF report

## Figures and Tables

**Table 1:** Hydrogen-bond geometry (Å, °)

*D*—H⋯*A*	*D*—H	H⋯*A*	*D*⋯*A*	*D*—H⋯*A*
C6—H6⋯O1^i^	0.95	2.55	3.151 (2)	121
C7—H7⋯O2	0.95	2.55	3.250 (2)	130
C12—H12⋯O2^ii^	0.95	2.51	3.435 (2)	165

Symmetry codes: (i) 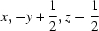; (ii) 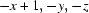.

**Fig. 1.**
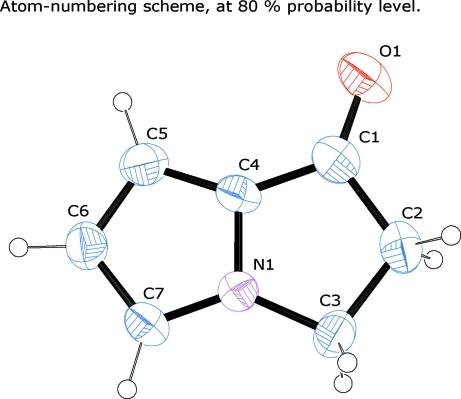
Displacement ellipsoid plot (80% probability level) showing atom numbering scheme.

**Fig. 2.**
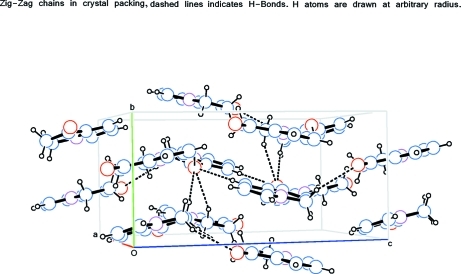
The packing showing the zigzg chains. Dashed lines indicate hydrogen bonds

## supplementary crystallographic information

### Comment

Derivatives of 2,3-dihydropyrrolizine became known through studies of their synthesis (Clemo *et al.*, 1931) and isolation from natural source (Meinwald *et al.*, 1965). Synthetic dihydropyrrolizines that are of interest as pharmaceuticals have been reported. The most important of these, Ketorolac, is a non steroid analgesic. Depending on their structure, derivatives of 2,3-dihydropyrrolizine have shown merit as analgesics, anti-inflammatory agents, myorelaxants, inhibitors of thrombocyte aggregation, fibrinolytics, temperature-lowering substances and drugs for the treatment of glaucoma and conjunctivitis (Skvortsov *et al.*, 1992).

The *ORTEP* (Farrugia, 1997) drawing of the molecule is shown in Fig. 1. The sums of the three angles at N1 and C4 are 359.93 and 359.96 respectively, indicating that two rings are almost planer with an r.m.s. deviation of 0.05 Å. Molecules are held together in crystal packing by weak C—H···O hydrogen bonds (Table 1), in the form of zigzag infinite one dimensional polymeric chains (Fig. 2.).

### Experimental

The preparation of title compound was carried out as described in the procedure reported in literature (Braunholtz *et al.*, 1962). Purified by Flash Column Chromatography, Petroleum Ether:Ethyl Acetate = 3:1.

### Refinement

All H atoms were placed in geometrically idealized positions and constrained to ride on their parent atoms with C—H = 0.95 and 0.99Å for aromatic and methylene respectively. *U*_iso_(H) values were taken to be equal to 1.2 *U*_eq_(C) for all hydrogen atoms.

### Crystal data

**Table:**

C_7_H_7_NO	*F*(000) = 512
*M**_r_* = 121.14	*D*_x_ = 1.381 Mg m^−^^3^
Monoclinic, *P*2_1_/*c*	Melting point: 327(1) K
Hall symbol: -P 2ybc	Mo *K*α radiation, λ = 0.71075 Å
*a* = 11.301 (1) Å	Cell parameters from 3403 reflections
*b* = 7.1730 (7) Å	θ = 1.8–28.1°
*c* = 14.3760 (16) Å	µ = 0.09 mm^−^^1^
β = 90.989 (5)°	*T* = 113 K
*V* = 1165.2 (2) Å^3^	Prism, colorless
*Z* = 8	0.12 × 0.06 × 0.04 mm

### Data collection

**Table:**

Rigaku Saturn724 CCD camera diffractometer	2284 independent reflections
Radiation source: rotating anode	2003 reflections with *I* > 2σ(*I*)
multilayer	*R*_int_ = 0.051
ω scans	θ_max_ = 26.0°, θ_min_ = 1.8°
Absorption correction: multi-scan (*CrystalClear*-SM Expert; Rigaku, 2009)	*h* = −13→13
*T*_min_ = 0.989, *T*_max_ = 0.996	*k* = −8→8
10183 measured reflections	*l* = −17→15

### Refinement

**Table:**

Refinement on *F*^2^	Primary atom site location: structure-invariant direct methods
Least-squares matrix: full	Secondary atom site location: difference Fourier map
*R*[*F*^2^ > 2σ(*F*^2^)] = 0.057	Hydrogen site location: inferred from neighbouring sites
*wR*(*F*^2^) = 0.118	H-atom parameters constrained
*S* = 1.16	*w* = 1/[σ^2^(*F*_o_^2^) + (0.043*P*)^2^ + 0.3505*P*] where *P* = (*F*_o_^2^ + 2*F*_c_^2^)/3
2284 reflections	(Δ/σ)_max_ < 0.001
163 parameters	Δρ_max_ = 0.19 e Å^−^^3^
0 restraints	Δρ_min_ = −0.30 e Å^−^^3^

### Special details

**Table:**

Experimental. Single crystals suitable for X-ray crystallography were grown by slow cooling of a hot saturated solution of Petroleum Ether.
Geometry. All e.s.d.'s (except the e.s.d. in the dihedral angle between two l.s. planes) are estimated using the full covariance matrix. The cell e.s.d.'s are taken into account individually in the estimation of e.s.d.'s in distances, angles and torsion angles; correlations between e.s.d.'s in cell parameters are only used when they are defined by crystal symmetry. An approximate (isotropic) treatment of cell e.s.d.'s is used for estimating e.s.d.'s involving l.s. planes.
Refinement. Refinement of *F*^2^ against ALL reflections. The weighted RSHELXS-97 -factor *wR* and goodness of fit *S* are based on *F*^2^, conventional *R*-factors *R* are based on *F*, with *F* set to zero for negative *F*^2^. The threshold expression of *F*^2^ > σ(*F*^2^) is used only for calculating *R*-factors(gt) *etc*. and is not relevant to the choice of reflections for refinement. *R*-factors based on *F*^2^ are statistically about twice as large as those based on *F*, and *R*- factors based on ALL data will be even larger.

### Fractional atomic coordinates and isotropic or equivalent isotropic displacement parameters (Å^2^)

**Table:**

	*x*	*y*	*z*	*U*_iso_*/*U*_eq_	
O1	0.84120 (12)	0.0832 (2)	0.57308 (9)	0.0293 (4)	
O2	0.49964 (11)	0.0828 (2)	0.17318 (10)	0.0329 (4)	
N1	0.72173 (13)	0.1521 (2)	0.35032 (11)	0.0181 (4)	
N2	0.20229 (13)	0.1557 (2)	0.11650 (11)	0.0190 (4)	
C1	0.78107 (17)	0.0987 (3)	0.50200 (13)	0.0214 (4)	
C2	0.64640 (16)	0.0776 (3)	0.49734 (13)	0.0229 (5)	
H2A	0.6086	0.1646	0.5413	0.027*	
H2B	0.6233	−0.0513	0.5136	0.027*	
C3	0.60795 (16)	0.1231 (3)	0.39650 (13)	0.0222 (4)	
H3A	0.5634	0.0183	0.3680	0.027*	
H3B	0.5586	0.2371	0.3938	0.027*	
C4	0.81910 (16)	0.1386 (3)	0.40853 (13)	0.0186 (4)	
C5	0.91883 (17)	0.1737 (3)	0.35603 (13)	0.0226 (4)	
H5	0.9988	0.1728	0.3775	0.027*	
C6	0.87834 (16)	0.2109 (3)	0.26502 (13)	0.0218 (4)	
H6	0.9265	0.2405	0.2136	0.026*	
C7	0.75535 (16)	0.1968 (3)	0.26317 (13)	0.0209 (4)	
H7	0.7046	0.2152	0.2106	0.025*	
C8	0.39292 (16)	0.1181 (3)	0.16955 (14)	0.0219 (4)	
C9	0.32016 (16)	0.1840 (3)	0.25181 (13)	0.0223 (4)	
H9A	0.3490	0.3065	0.2743	0.027*	
H9B	0.3260	0.0934	0.3036	0.027*	
C10	0.19132 (16)	0.1993 (3)	0.21584 (13)	0.0208 (4)	
H10A	0.1395	0.1084	0.2472	0.025*	
H10B	0.1596	0.3265	0.2250	0.025*	
C11	0.31432 (15)	0.1065 (3)	0.09001 (13)	0.0186 (4)	
C12	0.30999 (17)	0.0643 (3)	−0.00456 (14)	0.0223 (4)	
H12	0.3741	0.0258	−0.0419	0.027*	
C13	0.19207 (17)	0.0901 (3)	−0.03344 (14)	0.0239 (5)	
H13	0.1615	0.0721	−0.0948	0.029*	
C14	0.12721 (17)	0.1468 (3)	0.04322 (14)	0.0238 (5)	
H14	0.0450	0.1741	0.0435	0.029*	

### Atomic displacement parameters (Å^2^)

**Table:**

	*U*^11^	*U*^22^	*U*^33^	*U*^12^	*U*^13^	*U*^23^
O1	0.0349 (8)	0.0343 (9)	0.0185 (8)	0.0044 (6)	−0.0039 (6)	−0.0005 (6)
O2	0.0195 (7)	0.0467 (10)	0.0324 (9)	0.0055 (6)	−0.0011 (6)	−0.0079 (7)
N1	0.0169 (7)	0.0209 (8)	0.0165 (8)	0.0002 (6)	0.0003 (6)	−0.0004 (7)
N2	0.0187 (8)	0.0195 (9)	0.0188 (9)	0.0013 (6)	0.0020 (7)	−0.0012 (7)
C1	0.0263 (10)	0.0175 (10)	0.0203 (11)	0.0021 (8)	−0.0006 (8)	−0.0020 (8)
C2	0.0280 (10)	0.0213 (10)	0.0196 (10)	−0.0017 (8)	0.0044 (8)	0.0000 (8)
C3	0.0179 (9)	0.0247 (10)	0.0241 (11)	−0.0019 (8)	0.0032 (8)	0.0006 (8)
C4	0.0198 (9)	0.0198 (10)	0.0160 (10)	0.0012 (7)	−0.0031 (8)	−0.0022 (8)
C5	0.0189 (9)	0.0254 (10)	0.0233 (11)	−0.0002 (8)	−0.0016 (8)	−0.0033 (9)
C6	0.0224 (10)	0.0237 (10)	0.0193 (10)	−0.0013 (8)	0.0032 (8)	−0.0003 (8)
C7	0.0238 (10)	0.0227 (10)	0.0161 (10)	0.0015 (8)	−0.0015 (8)	0.0016 (8)
C8	0.0207 (9)	0.0200 (10)	0.0249 (11)	−0.0004 (8)	0.0007 (8)	0.0004 (8)
C9	0.0230 (10)	0.0251 (10)	0.0187 (10)	−0.0004 (8)	0.0007 (8)	−0.0008 (8)
C10	0.0222 (10)	0.0228 (10)	0.0177 (10)	0.0015 (8)	0.0043 (8)	−0.0020 (8)
C11	0.0190 (9)	0.0183 (10)	0.0184 (10)	0.0016 (7)	0.0030 (8)	0.0000 (8)
C12	0.0267 (10)	0.0199 (10)	0.0204 (10)	0.0004 (8)	0.0039 (8)	−0.0003 (8)
C13	0.0307 (11)	0.0233 (11)	0.0176 (10)	0.0011 (8)	−0.0024 (8)	0.0005 (8)
C14	0.0222 (10)	0.0244 (11)	0.0246 (11)	0.0008 (8)	−0.0044 (8)	0.0001 (9)

### Geometric parameters (Å, °)

**Table:**

O1—C1	1.222 (2)	C5—H5	0.9500
O2—C8	1.232 (2)	C6—C7	1.393 (2)
N1—C7	1.354 (2)	C6—H6	0.9500
N1—C4	1.374 (2)	C7—H7	0.9500
N1—C3	1.472 (2)	C8—C11	1.438 (3)
N2—C14	1.343 (2)	C8—C9	1.527 (3)
N2—C11	1.374 (2)	C9—C10	1.541 (3)
N2—C10	1.469 (2)	C9—H9A	0.9900
C1—C4	1.446 (3)	C9—H9B	0.9900
C1—C2	1.530 (3)	C10—H10A	0.9900
C2—C3	1.541 (3)	C10—H10B	0.9900
C2—H2A	0.9900	C11—C12	1.393 (3)
C2—H2B	0.9900	C12—C13	1.401 (3)
C3—H3A	0.9900	C12—H12	0.9500
C3—H3B	0.9900	C13—C14	1.395 (3)
C4—C5	1.390 (3)	C13—H13	0.9500
C5—C6	1.404 (3)	C14—H14	0.9500
			
C7—N1—C4	110.21 (16)	N1—C7—C6	107.17 (17)
C7—N1—C3	135.39 (16)	N1—C7—H7	126.4
C4—N1—C3	114.33 (15)	C6—C7—H7	126.4
C14—N2—C11	110.07 (16)	O2—C8—C11	127.76 (18)
C14—N2—C10	135.23 (16)	O2—C8—C9	124.78 (18)
C11—N2—C10	114.68 (15)	C11—C8—C9	107.47 (15)
O1—C1—C4	128.66 (18)	C8—C9—C10	106.29 (15)
O1—C1—C2	124.46 (18)	C8—C9—H9A	110.5
C4—C1—C2	106.88 (16)	C10—C9—H9A	110.5
C1—C2—C3	106.52 (15)	C8—C9—H9B	110.5
C1—C2—H2A	110.4	C10—C9—H9B	110.5
C3—C2—H2A	110.4	H9A—C9—H9B	108.7
C1—C2—H2B	110.4	N2—C10—C9	102.47 (14)
C3—C2—H2B	110.4	N2—C10—H10A	111.3
H2A—C2—H2B	108.6	C9—C10—H10A	111.3
N1—C3—C2	102.70 (14)	N2—C10—H10B	111.3
N1—C3—H3A	111.2	C9—C10—H10B	111.3
C2—C3—H3A	111.2	H10A—C10—H10B	109.2
N1—C3—H3B	111.2	N2—C11—C12	108.01 (16)
C2—C3—H3B	111.2	N2—C11—C8	108.92 (16)
H3A—C3—H3B	109.1	C12—C11—C8	143.08 (18)
N1—C4—C5	107.75 (16)	C11—C12—C13	106.13 (17)
N1—C4—C1	109.38 (16)	C11—C12—H12	126.9
C5—C4—C1	142.83 (17)	C13—C12—H12	126.9
C4—C5—C6	106.62 (16)	C14—C13—C12	108.33 (17)
C4—C5—H5	126.7	C14—C13—H13	125.8
C6—C5—H5	126.7	C12—C13—H13	125.8
C7—C6—C5	108.24 (17)	N2—C14—C13	107.47 (17)
C7—C6—H6	125.9	N2—C14—H14	126.3
C5—C6—H6	125.9	C13—C14—H14	126.3
			
O1—C1—C2—C3	175.74 (18)	O2—C8—C9—C10	−176.94 (19)
C4—C1—C2—C3	−4.4 (2)	C11—C8—C9—C10	3.2 (2)
C7—N1—C3—C2	−179.46 (19)	C14—N2—C10—C9	−178.40 (19)
C4—N1—C3—C2	−2.6 (2)	C11—N2—C10—C9	3.8 (2)
C1—C2—C3—N1	4.14 (19)	C8—C9—C10—N2	−4.05 (19)
C7—N1—C4—C5	−0.8 (2)	C14—N2—C11—C12	0.0 (2)
C3—N1—C4—C5	−178.45 (15)	C10—N2—C11—C12	178.29 (15)
C7—N1—C4—C1	177.53 (15)	C14—N2—C11—C8	179.72 (15)
C3—N1—C4—C1	−0.1 (2)	C10—N2—C11—C8	−2.0 (2)
O1—C1—C4—N1	−177.28 (18)	O2—C8—C11—N2	179.24 (19)
C2—C1—C4—N1	2.8 (2)	C9—C8—C11—N2	−0.9 (2)
O1—C1—C4—C5	0.1 (4)	O2—C8—C11—C12	−1.2 (4)
C2—C1—C4—C5	−179.7 (2)	C9—C8—C11—C12	178.7 (2)
N1—C4—C5—C6	0.7 (2)	N2—C11—C12—C13	0.1 (2)
C1—C4—C5—C6	−176.7 (2)	C8—C11—C12—C13	−179.6 (2)
C4—C5—C6—C7	−0.4 (2)	C11—C12—C13—C14	−0.1 (2)
C4—N1—C7—C6	0.6 (2)	C11—N2—C14—C13	0.0 (2)
C3—N1—C7—C6	177.51 (19)	C10—N2—C14—C13	−177.84 (19)
C5—C6—C7—N1	−0.1 (2)	C12—C13—C14—N2	0.0 (2)

### Hydrogen-bond geometry (Å, °)

**Table:**

*D*—H···*A*	*D*—H	H···*A*	*D*···*A*	*D*—H···*A*
C6—H6···O1^i^	0.95	2.55	3.151 (2)	121
C7—H7···O2	0.95	2.55	3.250 (2)	130
C12—H12···O2^ii^	0.95	2.51	3.435 (2)	165

Symmetry codes: (i) *x*, −*y*+1/2, *z*−1/2; (ii) −*x*+1, −*y*, −*z*.
